# The role of constructive patriotism in the relationship of basic human values and active citizenship for emerging adults in Türkiye

**DOI:** 10.1186/s40359-023-01233-z

**Published:** 2023-07-12

**Authors:** Mehmet Elban, Selçuk Aslan

**Affiliations:** 1 Faculty of Education, Department of Social Science Education, Fırat University, Elazığ, Türkiye; 2 Medical Center, PCGC/Psychiatry Unit, Middle East Technical University, Ankara, Türkiye

**Keywords:** Active citizenship, Constructive and blind patriotism, Basic human values, Individual and collectivist values

## Abstract

Active citizenship is closely related to basic human values and patriotism, but empirical studies have lacked investigating these variables holistically. Likewise, the mediating role of patriotism between basic human values and active citizenship seems neglected. In this context, the current study examined the relationships between basic human values, patriotism, and active citizenship by grounding upon basic human values, individualism, and collectivism theories. Results revealed that constructive patriotism positively predicted all dimensions of active citizenship (political literacy, participation and protest, and social responsibility) and mediated the relationships between basic human values and active citizenship. Additionally, while stimulation and self-direction individual values, including motivational goals such as challenges in life, independent thought, and autonomy, were associated with the individual aspect of active citizenship (protest and individual responsibility), the values based on equality (benevolence) were related with the collectivist aspect of active citizenship (participation and social responsibility). One of the most influential findings was that the universalism value could only associate positively with active citizenship through constructive patriotism. Another impressive culture-specific result was that the political literacy dimension of active citizenship was positively related to power, which includes the goal of achieving individual success and dominating others. It can be evaluated as Turkish culture is more of a vertical collectivist because, historically and sociologically, a way of gaining power and status has been possible with politics and its cognitive equivalent, political literacy. The study also revealed that active citizenship is promoted through individual values based upon an individual’s independent choices and collectivist values that support societal interests based on equality. In summary, the research mainly revealed that active citizenship is sustained by both individualist values (stimulation, self-direction) and collectivist values (universalism, benevolence), and constructive patriotism has a critical mediating role. Implications of the results were discussed with the relevant literature.

## Introduction

The world currently struggles with many vital problems like the COVID-19 pandemic [1], refugees and asylum seekers [2] insufficient water resources, climate change with global warming [3], global energy, food supply chains caused by the Russia-Ukraine war [4]. Such global problems that also determine the qualities of social trust, civil society, government-citizen relationship, social cohesion, and co-survival [1, 5] affect all countries both at the societal and individual level and concern all the citizens closely. Only a few governmental actors will unlikely solve these major problems by considering the common good. Citizens should also take individual initiative. The individual’s conscious and pragmatic striving for society to form a cohesive community and feeling of oneness and belonging seems crucial for both societal and personal well-being [6, 7]. Meaning-making as a distinctive human action [8] is closely related to this reciprocal relationship between the individual and society because the self is not an independent structure from other people [9]. Even humans’ evolutionary divergence from other species and survival is closely related to prosociality, including cooperation and communication [10]. Even biologically a social being, the individual acts towards his innate social interest by transcending himself, cooperating, contributing to society, and overcoming worldwide problems [11, 12]. Likewise, taking responsibility by feeling ownership and belonging to other people and the country can signify secure attachment with society. In this regard, active citizenship and constructive patriotism can also be defined as a kind of healthy attachment to society [13].

At a time when such global problems above are increasing, the type of citizenship that contributes to the solution of global problems can be active citizenship. The reason is that active citizenship incorporates sufficiencies and active efforts to solve the problems mentioned above. These efficacies and activities include features such as collecting information, utilizing their constitutional rights, and speaking freely [14], civic and political participation, a culture of protest, respect for different groups, and responsibility [15–17]. However, why are some individuals active citizens and some not? Indeed, many factors affect being an active citizen.

The sub-dimensions of active citizenship encompass the prerequisites for being an active citizen. One crucial sub-dimension is political literacy, which entails three common elements found in various definitions: “cognitive, attitude/affective, and behavioral aspect”. The cognitive aspect involves acquiring information about the political situations surrounding the individual. The attitude/affective aspect refers to an individual’s attitudes and beliefs regarding political situations. The behavioral aspect dimension is political behavior motivated by knowledge, attitudes, and beliefs. Behaviors such as expressing political thoughts, participating in a specific political action such as an election, supporting or criticizing the ruling government, and surveilling public services are the behavioral aspects of political literacy [18]. Studies reveal that political literacy self-efficacy is an essential prerequisite for active citizenship [19, 20]. The level of political literacy and, consequently, citizenship education are related to individuals’ belief in their ability to create change and their values [20–22]. In addition, one of the active citizenship self-efficacies idealized by the Crick Report [23] is political literacy [24]. However, research into political literacy is still inadequate, and new research needs in that area [25].

Another essential dimension of active citizenship is self-efficacy of participation/engagement. According to the related theory, civic participation/engagement is a form or sub-dimension of “active citizenship.“ [26]. Here we mean civic participation/engagement rather than political participation/engagement. Civic engagement/participation refers to an individual’s voluntary efforts to improve the lives of others and create a good community or society and make the community or society a better place to reside. These individual efforts may be of an individual or collective type [27, 28]. Related research highlights the need to understand individuals’ self-efficacy of civic participation/engagement to become active citizens [29–31].

The concept accompanying civic participation/engagement is a social or civic responsibility. Social responsibility means a feeling of responsibility for others and an affective sense of connection to others in the community (empathy). Another concept very closely related to social responsibility is civil responsibility [32]. With a focus on the common good, civic responsibility means active participation in the community’s public life in an informed, committed, and constructive manner [33]. Social responsibility is a higher concept that includes civil responsibility [34]. Social and civic responsibility is considered as a prerequisite for being an active citizen [35–37]. It has been taken place that creating environments that promote social responsibility is particularly necessary for college and university settings [38]. Thus, we can say that understanding the factors that promote social and civic responsibility is a crucial way to understand the self-efficacies of active citizenship.

Another dimension accompanying social/civic responsibility is the protest self-efficacy of active citizens. Moreover, protest activities are considered one of the main indicators of active citizenship [39, 40]. According to some studies, protest activities are a form of political participation/engagement [39, 41]. In addition, protest activities are also evaluated as civic and political-oriented [42]. Thus, active citizenship activities are generally divided into two political and community oriented. These action types are defined as civil society action. In this context, protests and demonstrations can be politically or community oriented [43]. There are also more popular protest behaviors by young citizens, such as “wearing or displaying a badge or sticker and participating in a demonstration” [44]. Understanding the factors that promote protest activities, whether they are political-oriented or community/civic-oriented, will contribute to understanding active citizenship. Moreover, previous research has revealed that further research is needed on protest intentions in terms of university youth, different cultures, and multiple contexts [39, 41].

Another important factor for active citizenship is values. Because values constitute another important dimension of citizenship education along with knowledge and skills [45, 46]. Values correspond to active existence in life [47]. As a matter of fact, western countries have seen democratic values as a vital part of democratic citizenship education [48, 49]. In particular, values such as freedom, democracy, tolerance, and respect for diversity [50], more inclusively, humanitarian and social values [47] are central to idealized citizenship. Previous studies on values and active citizenship or its sub-dimensions have revealed some of this structure. Political participation, protest and engagement, which is an indicator of active citizenship, is closely related to basic human values [51, 52]. For example, self-transcendence values (universalism and benevolence) promote political participation [51, 53]. Likewise, positive relationships were found between self-transcendence values and civic participation, equality, civil liberties, and community activity [53–57]. Like self-transcendence values, openness-to-change values also have positive relationships with political participation [55, 58]. Also, previous studies have shown that the types of global citizenship (Political, economic, spiritual, etc.) associated with active citizenship are most positively correlated with openness to change values and self-transcendence values [59]. On the other hand, self-enhancement and conservation values were generally negatively related to the dimensions of active citizenship [55, 57].

That said, basic human values are not only associated with citizenship self-efficacies but also with types of patriotism. Previous studies have indicated positive correlations between self-transcendence values (universalism, benevolence) and openness-to-change values (stimulation, self-direction), and constructive patriotism [55, 60, 61]. When citizenship self-efficacy was taken into account, it is seen that patriotism has a strong relationship with active citizenship self-efficacy. According to previous studies, constructive patriotism, a democratic, critical citizenship type, has positive relations with civic and political participation or related citizenship types (Global), multicultural policy support, and community action, which is the dimension of active citizenship. Uncritical citizenship-type blind patriotism is negatively related to the dimensions of active citizenship [59, 62–68]. We observe that the conducted research reveals the empirical relationship between patriotism and participation [69]. Because civic patriotism inherently reflects democratic citizenship traits such as government accountability and political engagement [69, 70]. More specifically, patriotism and social responsibility come together in young people’s voluntary civic participation and engagement activities [71]. To put it differently, patriotism can promote an individual’s active participation for the well-being of their community and the well-being of other communities [72]. For instance, constructive patriotism encourages self-efficacy in gathering information/political literacy, political information gathering, political knowledge, and political involvement. On the other hand, blind patriotism is positively associated with political disengagement and political ignorance [65, 73, 74]. The compatibility of constructive/critical patriotism with critical and change-oriented actions is the reason for this [73, 74]. Hence the strong relationship of patriotism between values and citizenship self-efficacies points to its mediating role between these variables. Indeed, some research results have shown that patriotism mediates between similar variables, such as values and citizenship self-efficacy. For instance, patriotism has been found to play a significant mediating role between political ideology and donation bias [75], religiosity and taxpayer compliance [76], and official media and confidence in the system [77].

Thus, it is crucial to answering the questions of how political and civic participation interacts with patriotism and what type of it [63, 65] and to what extent the constructive dimension of patriotism is important in predicting civic participation [78]. In addition, if participatory citizenship is an important part of democracy, it is necessary to understand the place of patriotism in this structure. The questions of whether patriotism is beneficial for democracy or does adherence to patriotism threaten democracy are also controversial issues that educators cannot agree on [17].

Although active citizenship includes multiple dimensions, in most studies, not all dimensions of active citizenship have been examined as a whole. The studies mostly test the relationships between basic human values and political and civic activity/participation of active citizenship [51, 53–55, 57, 58, 79]. In addition, previous studies have examined the relations between the dimensions of citizenship, political and civic activity/participation, and types of patriotism without consideration of basic human values [63, 65, 78, 80]. Some studies focused more on the relations between types of patriotism and basic human values without consideration of active citizenship [60, 61, 81]. We still lack knowledge of the relationships between basic human values, patriotism, and active citizenship. Also, the mediating role of patriotism between values and active citizenship has yet to be examined. The current research contributes to the literature by revealing the direct and indirect effects of basic human values and types of patriotism on active citizenship in order to fill this gap. In addition, the current research has brought a new perspective to active citizenship in terms of showing which individual and collectivist values affect patriotism and active citizenship more.

Likewise, emerging adults constituted the current study sample. Arnett, conceptualized emerging adulthood as a distinct developmental period between the ages of 18–29 and characterized being between the confusions of adolescence and the responsibilities of adulthood, and families are still needed. Emerging adults experience delay and uncertainty in life tasks such as education, career, and marriage with enhancing effect of industrialization and technology. Exploration of identity, instability, focusing on self, in-betweenness, and optimism about possibilities are common defining features of this distinct span. Therefore, it is crucial to investigate active citizenship in emerging adults because they are demanding, and active citizenship requires them to be action-oriented in harmony with the community to reach their demands [82].

### Concepts

#### Active citizenship

There is no agreed common definition of active citizenship in the relevant literature [83]. However, the common elements for active citizenship are listed. Accordingly, active citizenship is a structure that includes civic and political participation, a culture of protest, respect for different groups, human rights, democratic participation, and responsibility [15–17]. It can be said that the concept of active citizenship has three dimensions: Affective, cognitive, and pragmatic. The affective dimension includes the individual’s attachment to society. In the cognitive dimension, there is confidence arising from knowledge. The pragmatic dimension includes the actions of the individual connected to previous dimensions [84]. In addition, the active citizen is expected to take an active part in at least one of the four areas, such as state / official politics, workplace, civil society, and private sphere, if possible, and be active in more [15].

#### Blind and constructive patriotism

Although patriotism expresses one’s loyalty to one’s country and is one of the types of national attachment, it is a concept that differs from national identity and nationalism [80]. When we look at the types of patriotism in the current research, it is seen that blind patriotism is an attachment-based on unconditional allegiance. So much so that this unconditional allegiance does not tolerate constructive criticism, even for the welfare of the country [65]. Put another way, blind patriotism is defined as not criticizing the policies of one’s country under any circumstances but simply showing loyalty to it [55]. On the other hand, constructive patriotism is the attachment to the country, which includes criticism and questioning of existing governmental/administrative practices for possible positive change [65]. The common point of constructive and blind patriotism is the love of the individual for own country. However, the active participation of these two types in political and civic life does not occur in the same way. Constructive patriots consider universality and human rights indispensable at the expense of group conformity, even taking into account the criticism of other patriots in the country. On the other hand, according to blind patriots, critical questioning and similar attitudes are incompatible with patriotism [17, 81]. It can be said that constructive patriotism, with its characters for democratic systems, is significantly intertwined with being a good citizen, and both are phenomena that define each other [60].

### Theoretical background

Psychologists, sociologists, and anthropologists studying values, which have different conceptual uses [85], have seen values as criteria that individuals use to evaluate people, actions, and events, including themselves [86]. As a continuation of this process, values function as axes that drive and direct one’s attitudes and behaviors [54, 86]. Values not only positively affect and contribute to individuals’ subjective well-being and sense of self-efficacy [87], but also form the basis of a sense of responsibility towards the society [54]. The most widely used theory of value was developed by Schwartz and Bilsky [88, 89]. According to this theory, there are primarily conscious goals or motivations in the content of values. Schwartz [86] revised the original theory and made some changes. Accordingly, the values consist of ten different values under four higher-order value types. Conservation values (security, tradition, and conformity) versus openness to change values (self-direction, and stimulation) describe the often unpredictable and independent choices and behaviors of a person towards one’s own intellectual and emotional interests. Conservation, on the other hand, includes certainty in social structures, traditions, and close relationships, and choices and behaviors aimed at preserving the status quo [86, 90]. Self-enhancement (hedonism, achievement, and power) versus self-transcendence (universalism and benevolence). Self-enhancement values are based on the individual’s taking care of his own interests, while self-transcendence values are based on the interests of the society in which the individual lives and even the whole humanity. Each of the values in the theory feeds on different motivational goals [55, 90].

Another theory accompanying Schwartz’s theory of values is the theory of individualism and collectivism. According to this theory, cultures differ as individual and collectivist [91–93]. In addition, based on this theory, both individualistic and collectivist cultural orientations are divided into horizontal and vertical. Accordingly, individualistic and collectivist horizontal cultures consider equality, individualistic and collectivist vertical cultures consider hierarchy and status [92, 93]. It is possible to see these four types of cultural orientations in all cultures [93]. Self-enhancement (power and achievement) is related to the vertical dimension. Self-transcendence (universalism and benevolence) is more associated with the horizontal dimension. On the vertical axis, the goals of the individual are related to hierarchy and status. In contrast, the horizontal axis includes goals based on equality [92–96]. Conservative values (tradition, conformity, security) and self-transcendence values (universalism, benevolence) is in a relationship with collective interest. In contrast, openness-to-change values (hedonism, stimulation, self-direction) and self-enhancement values (power, achievement) are associated with individual interests [86, 90]. However, values that serve individual goals and interests may gain priority in individual cultures, while values that serve collectivist goals and interests may gain more priority in collectivist cultures [86].

**Fig. 1:**
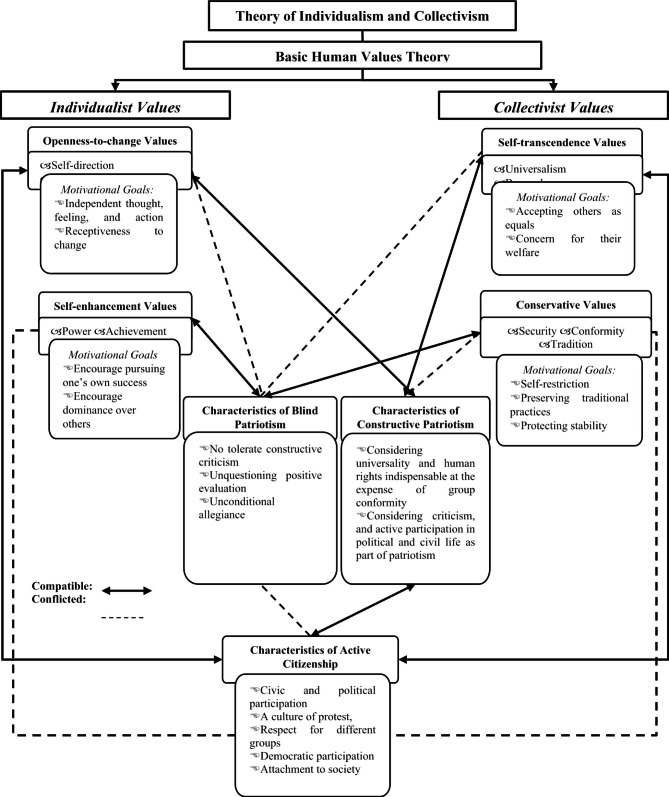
The Compatibility of basic human values and types of patriotism with active citizenship

Based on these theories, we can say that self-transcendence values (universalism, benevolence), which are based on the equality and welfare of all people and are more called horizontal collectivist values, are expected to positively affect the social responsibility dimension of active citizenship. Also, openness-to-change values (stimulation, self-direction), where independent decisions are at the forefront, are expected to positively affect protest and activism. It is expected that conservative values (tradition, conformity, and security) among the collectivist values regarding the preservation of the status quo negatively affect or do not affect the dimensions of active citizenship in which individual views and goals are at the forefront. The compatibility of basic human values and types of patriotism with active citizenship is given in Fig. 1.

### Predictors of active citizenship

It is crucial to study values in understanding the motivations behind civic and political actions, taking into account the impact of the social, cultural, and economic context [15, 58]. Also, the relationship between human values and civic engagement has been revealed [53, 79]. Universalism, benevolence, and stimulation can positively influence participation for others’ welfare [57, 58].Therefore, individuals with universalism, benevolence and stimulation values are expected to participate in organizations for their community or for all humanity.

#### H1

Universalism has a positive effect on participation.

#### H2

Benevolence has a positive effect on related to participation.

#### H3

Stimulation has a positive effect on participation.

The definition of active citizenship emphasizes the importance of values for participation [43]. Value priorities, one of the components of personality, have the power to influence the behavior of citizens, including political and civic activism. In other words, basic values suitable for behavioral expression can contribute to the civic and political actions of individuals and/or citizens [58]. In addition, basic human values (self-direction, universalism, and stimulation) are effective on individuals’ voting behavior. Fundamental values also have an impact on political activism as a broader concept than voting [56]. Nonetheless, active citizens tend towards social and humanitarian situations [79]. Thus, according to “the basic human values theory” and “the theory of individualism and collectivism,” basic values can be expected to affect active citizenship as follows:

#### H4

Self-direction has a positive effect on protest and social responsibility.

#### H5

Universalism has a positive effect on protest and social responsibility.

#### H6

Stimulation has a positive effect on protest and social responsibility.

Conservative values (tradition, conformity, security) are more related to right authoritarian ideologies [81, 97]. It is also stated that conservative values are negatively related to political activism [56, 58]. On the other hand, there are relations between the power values of individuals and political associationism [53]. Thus, it was predicted that tradition, conformity and security have a negative effect on political literacy. Also, we can say that power has a positive effect on political literacy.

#### H7

Tradition has a negative effect on political literacy.

#### H8

Conformity has a negative effect on political literacy.

#### H9

Security has a negative effect on political literacy.

#### H10

Power has a positive effect on political literacy.

It is seen that values have an effect on the types of patriotism. There are relations between constructive patriotism as a type of patriotism and active citizenship competencies [63–65]. For example, self-transcendence values rather than self-enhancement were expected to positively affect constructive patriotism. Because while in the first, there is the priority of the individual, in the second, there is the priority of the others or the well-being of the society and even the whole humanity [60]. Likewise, the values of universalism and self-direction that encourage tolerance towards others and different ideas [55] are in line with the nature of constructive patriotism. Therefore, it is possible that the values mentioned, and constructive patriotism will positively affect active citizenship. Put another way, there may be a mediating role of constructive patriotism. On the other hand, blind patriotism has no importance over active citizenship [65]. Also, it is stated that types of patriotism are also effective on civic and political participation, which are the dimensions of active citizenship [60, 81]. According to the related theory, the following hypotheses can be formed:

#### H11

Constructive patriotism has a positive effect on active citizenship.

#### H12

Blind patriotism has no effect on active citizenship.

#### H13

Self-direction has a positive effect on protest and social responsibiltiy via constructive patriotism.

#### H14

Self-direction has a positive effect on participation via constructive patriotism.

#### H15

Self-direction has a positive effect on political literacy via constructive patriotism.

#### H16

Universalism has a positive effect on protest and social responsibiltiy via constructive patriotism.

#### H17

Universalism has a positive effect on participation via constructive patriotism.

#### H18

Universalism has a positive effect on political literacy via constructive patriotism.

## Method

Predictive research is estimating dependent variables via independent variables [98]. Therefore, it can be said that the current research aiming to predict active citizenship with independent variables (Personal basic values and blind-constructive patriotism) is predictive research.

### Participants and procedure

Path analysis was used to test our hypothesis model. Power analysis was performed to determine the adequate sample size needed for this purpose. There are methods, such as the Monte Carlo method, to conduct power analysis in structural equation models. Another effective method for structural equation models and similar path models is RMSEA-based power calculations [99, 100]. In this context, RMSEA-based power calculation was made using “Shiny app power4SEM” for the sample size of the research. A minimum power of 80% is generally considered acceptable [101–103]. But, our goal was to obtain 0.95 power. To obtain a power of 0.95 (H_0_ RMSEA = 0.05, H_1_ RMSEA = 0.08, df = 246, and alpha = 0.05), power4SEM indicated a minimum sample size of 106. Data for analyses were collected from the students of a state university in Türkiye. The participants consist of 360 undergraduate students. Of the participants, 122 were male (33.9%), and 238 were female (66.1%). The ages of the participants vary between 18 and 26 (mean age ± standard deviation: 20.94 ± 1.82; age range: 18–26 years). The students in the research sample are studying in ten different departments. The students in the research sample studied eleven different departments (Primary school teaching department: 108 students 30%, guidance and psychological counseling department: 77 students 21.4%, social studies teaching department: 61 students 16.9%, Turkish language and literature department: 47 students 13.1%, banking department: 21 students 5.8%, public finance department: 16 students 4.4%, Turkish education department: 15 students 4.2%, postal service department: 9 students 2.5%, business management department: 3 students 0.8%, elementary mathematics teaching department: 2 students 0.6% and cookery department: 1 students 0.3%).

The current study was conducted in accordance with the guidelines proposed in the Declaration of Helsinki. Also, the ethics committee of Bayburt University approved the research (The committee’s reference number: 01.29.2021, E-79126184-050.99-3831). The data were collected from the university where the corresponding author worked but from different faculties and departments where the corresponding author did not attend students’ courses. The collection of data was based on voluntariness. The corresponding author received assistance from his colleagues in announcing and filling out the questionnaires to the students. According to the ethical approval, it was stated that participants under eighteen years old could not participate in the survey even if they volunteered. Participants were asked about their demographic characteristics, such as age, gender, and the department they studied. Participants were not asked to write their names and surnames on the questionnaires. It was stated to the participants that when they felt uncomfortable with the questions, they could stop filling out the scales at any time. Five students did not want to participate in the volunteer-based study. Ten participants did not fill in more than half of their forms. Therefore, these forms were not processed.

## Measures

### Patriotism attitude scale (PAS)

The Patriotism Attitude Scale was developed by Schatz et al. [65]. The scale was adapted into Turkish by Yazıcı and Yazıcı [104]. PAS were scored on a 5-point Likert type-scale, from 1 to 5 representing “totally disagree” to “totally agree”, respectively. The scale consists of two dimensions. The first dimension measures constructive patriotic attitudes, and the second dimension measures blind patriotic attitudes. PAS contains twenty questions. The example questions were as follows: “If I criticize Türkiye, I do so out of love for my country (Constructive patriotism),” and “There is too much criticism of Türkiye in the world, and we its citizens should not criticize it (Blind patriotism)”. The internal consistency coefficient was found to be 0.78 for blind patriotism and 0.74 for constructive patriotism.

### Portrait values questionnaire (PVQ)

This questionnaire was developed by Schwartz et al. [90] and adapted into Turkish by Demirutku and Sümer [105]. The 40-item scale measures value types [90]. The scale consists of ten value types. PVQ were scored on a 6-point Likert type-scale, from 1 (not like me at all) to 6 (very much like me). The example questions were as follows: “He always wants to be the one who makes the decisions. He likes to be the leader (Power)”, “Getting ahead in life is important to him. He strives to do better than others (Achievement)”, “He seeks every chance he can to have fun. It is important to him to do things that give him pleasure (Hedonism)”, “He likes to take risks. He is always looking for adventures (Stimulation)”, “It is important to him to make his own decisions about what he does. He likes to be free to plan and to choose his activities for himself (Self-Direction)”, “He believes all the worlds’ people should live in harmony. Promoting peace among all groups in the world is important to him (Universalism)”, “It’s very important to him to help the people around him. He wants to care for their well-being (Benevolence)”, He thinks it is best to do things in traditional ways. It is important to him to keep up the customs he has learned (Tradition)”, “He believes that people should do what they’re told. He thinks people should follow rules at all times, even when no one is watching (Conformity)”, and “It is very important to him that his country be safe. He thinks the state must be on watch against threats from within and without (Security)”. The Cronbach alpha internal consistency coefficients of the subscales were 0.60, 0.69, 0.61, 0.60, 0.64, 0.74, 0.66, 0.64, 0.71, and 0.63 for power, achievement, hedonism, stimulation, self-direction, universalism, benevolence, tradition, conformity, and security, respectively.

### Active citizenship self-efficacy scale (ACSES)

The scale was developed in Turkish culture by Arslan et al. [106]. The scale consists of eighteen items and three subscales (Political literacy, participation, protest and social responsibility). The measurement tool measures the active citizenship self-efficacy levels of individuals. ACSES were rated on a 5-point Likert type-scale, from 1 to 5 representing “totally disagree” to “totally agree”, respectively. The example questions were as follows: “I can protest something that I see as unfair (Protest and social responsibility)”, “I can comprehend what is happening in politics (Political literacy)”, and “I can readily participate in activities organized by an association, foundation, or others (Participation)”. In the current study, Cronbach alpha internal consistency coefficient was 0.88 for the entire scale. Cronbach alpha internal consistency coefficient was 0.84, 0.80, and 0.82 for political literacy, participation, protest and social responsibility, respectively.

### Data analysis

Descriptive statistics and correlation analysis of the research data were calculated with SPSS 23.0. Research hypotheses were tested with path analysis from structural equation modeling. We used AMOS 20.0 to estimate the direct and indirect paths. Since the data set of the research consists of (n = 360) participants, it can be said that the sample number meets the structural modeling assumptions. Mahalanobis distance values were examined to determine the multivariate extreme values of the research data. In addition, the absence of a correlation greater than 0.90 between the research variables indicates that there is no multicollinearity problem [107]. The univariate and multivariate normality of the data was examined. Since the skewness values of the data are between − 0.804 and − 0.175, and the kurtosis values are between − 0.502 and 0.153, the univariate normality assumption is provided [108]. On the other hand, since the critical ratio value of the data (c.r.=1.913) is less than 10, the research data provides the assumption of multivariate normality [109]. The bootstrapping method (5.000 resampling) was used to examine the mediating effect of the variables in the model. A range of statistical indices was employed to evaluate the model’s goodness of fit, including χ2/df, GFI, AGFI, NNFI, CFI, TLI, RMSEA, and SRMR. The following critical values were taken into account in defining the acceptance point: 2 < χ2 /df < 5 [110], GFI ≥ 0.90 or 0.95, AGFI ≥ 0.90 or 0.95, NNFI/TLI ≥ 0.90 or 0.95, RMSEA ≤ 0.05 to 0.08 [111], CFI ≥ 0.90 [112], and SRMR < 0.08 [113].

## Results

### Descriptive and correlation analysis

Table 1 presents the means, standard deviations, score ranges and correlations of variables. The highest value type average of the participants in the sample was universalism (mean 5.43, *SD* 0.49), and the lowest value type average was power (mean 3.93, *SD* 1.00).

**Table 1:** Descriptive statistics and correlation coefficients of all variables used in the study

Variables	1	2	3	4	5	6	7	8	9	10	11	12	13	14	15
Power	1														
Achievement	0.550^**^	1													
Hedonism	0.309^**^	0.327^**^	1												
Stimulation	0.236^**^	0.304^**^	0.409^**^	1											
Self-direction	0.232^**^	0.363^**^	0.236^**^	0.411^**^	1										
Universalism	-0.069	0.083	0.214^**^	0.243^**^	0.388^**^	1									
Benevolence	-0.080	0.144^**^	0.179^**^	0.227^**^	0.248^**^	0.524^**^	1								
Tradition	-0.160^**^	-0.015	-0.004	0.120^*^	0.103	0.299^**^	0.384^**^	1							
Conformity	0.016	0.145^**^	0.093	0.189^**^	0.148^**^	0.398^**^	0.461^**^	0.471^**^	1						
Security	0.096	0.239^**^	0.183^**^	0.203^**^	0.329^**^	0.366^**^	0.296^**^	0.307^**^	0.400^**^	1					
Political literacy	0.175^**^	0.132^*^	0.020	0.059	0.154^**^	0.073	-0.065	-0.165^**^	-0.070	0.048	1				
Participation	0.017	0.129^*^	0.195^**^	0.297^**^	0.272^**^	0.368^**^	0.339^**^	0.083	0.118^*^	0.137^**^	0.271^**^	1			
Protest and social responsibiltiy	0.138^**^	0.208^**^	0.231^**^	0.299^**^	0.360^**^	0.278^**^	0.163^**^	0.051	0.070	0.183^**^	0.397^**^	0.641^**^	1		
Blind	0.056	0.159^**^	-0.025	0.113^*^	0.029	-0.068	0.075	0.262^**^	0.151^**^	0.231^**^	0.033	-0.087	-0.011	1	
Constructive	0.057	0.110^*^	0.102	0.057	0.250^**^	0.292^**^	0.202^**^	0.051	0.133^*^	0.144^**^	0.219^**^	0.294^**^	0.280^**^	-0.054	1
*M*	3.93	4.47	4.90	4.79	5.22	5.43	5.17	4.69	5.05	5.27	3.27	3.76	3.79	3.10	4.28
*SD*	1.00	0.87	0.84	0.73	0.55	0.49	0.64	0.74	0.68	0.53	0.81	0.72	0.63	0.58	0.43
Score Range	1.00–6.00	1.75-6.00	2.33–4.90	2.67-6.00	3.50-6.00	3.50-6.00	3.00–6.00	2.50-6.00	3.00–6.00	3.60-6.00	1.00–5.00	1.80-5.00	2.11-5.00	1.67–4.67	2.88-5.00

Participants had the highest mean score on the protest and social response dimension of active citizenship (mean 3.79, *SD* 0.63) and the lowest score on the political literacy dimension (mean 3.27, *SD* 0.81). Participants’ average blind patriotism (mean 3.10, *SD* 0.58) was lower than their constructive mean score (mean 4.28, *SD* 0.43). All dimensions of active citizenship were not significantly correlated with blind patriotism (r = 0.33, -0.09, -0.01; p < 0.05). On the other hand, the political literacy dimension (r = 0.22, p < 0.01), participation dimension (r = 0.29, p < 0.01), and protest and social responsibility dimension (r = 0.28, p < 0.01) of active citizenship were significantly and positively correlated with constructive patriotism. When the relationships between values and the dimensions of active citizenship were examined, there were positive correlations between political literacy and power (r = 0.16, p < 0.01) and achievement (r = 0.13, p < 0.05). Political literacy significantly negatively correlated with tradition (r=-0.17, p < 0.01). The positive and significant relationships between the ten value types and the three sub-dimensions of active citizenship ranged from 0.02 to 0.37. Self-direction, universalism, and benevolence were the value types that showed the highest positive correlation with the dimensions of active citizenship. Thus, the hypotheses were initially tested.

### Path analysis and non-significant paths

As can be seen in Table 1., blind patriotism was not correlated with active citizenship. Likewise, the effect of blind patriotism on the sub-dimensions of active citizenship was insignificant (political literacy *β* = 0.03, *p* > 0.05; participation *β* = -0.09, *p* > 0.05; protest and social responsibility *β* = -0.04, *p* > 0.05). Therefore, blind patriotism was excluded from the model (H11.was rejected). In the first analysis, security and conformity did not predict the political literacy dimension of active citizenship (security→ political literacy *β* = 0.07, *p* > 0.05; conformity→ political literacy; *β*= -0.03, p > 0.05). Therefore, security and conformity were excluded from the model (H8. and H9. were rejected). Universalism did not directly predict participation and protest and responsibility, two sub-dimensions of active citizenship (universalism→ participation *β* = 0.09, *p* > 0.05; universalism →protest and social responsibility; *β* = 0.11, *p* > 0.05). (H1. and H5. were rejected).

### The final path model

**Fig. 2:**
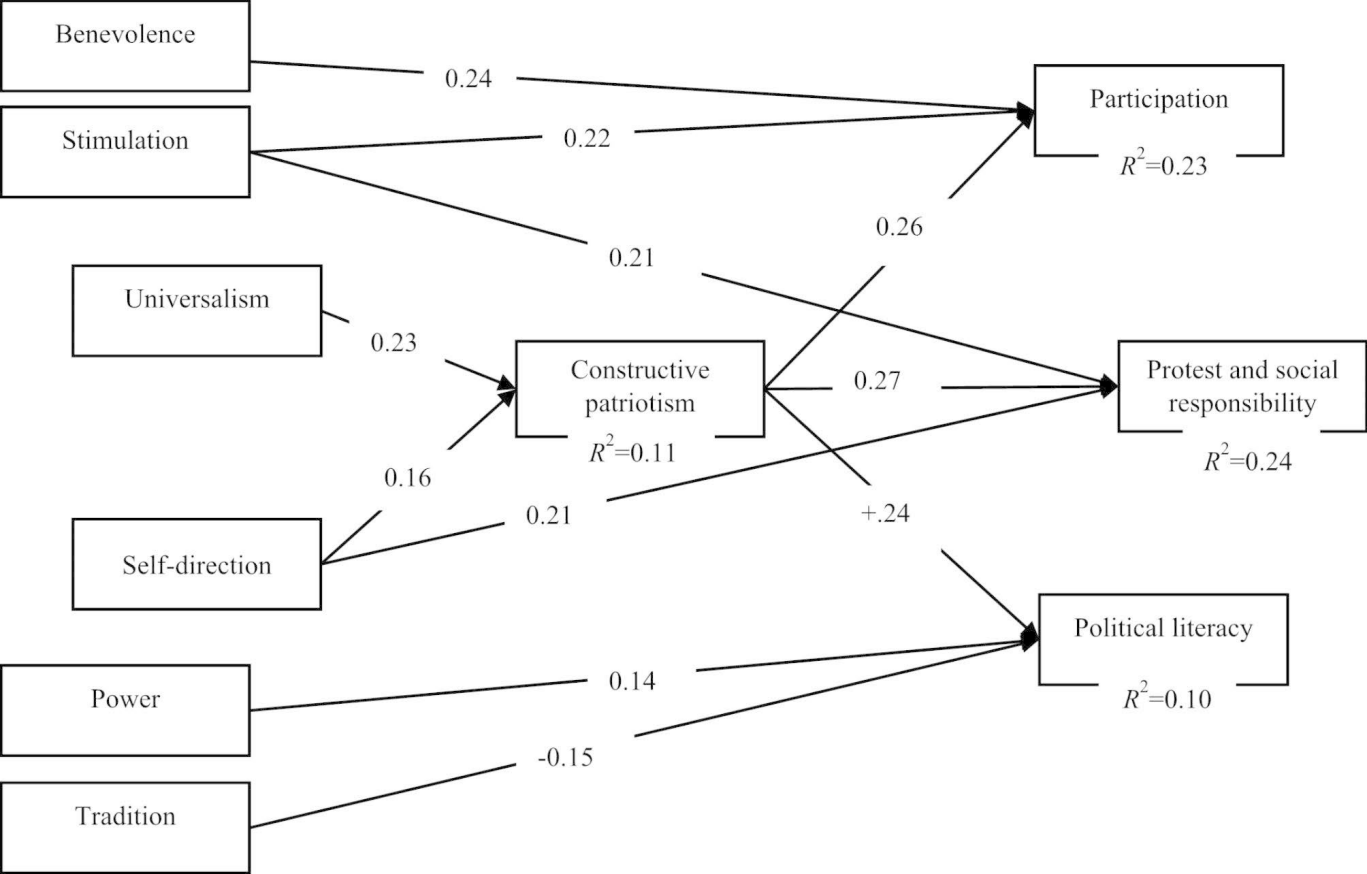
The final path diagram regarding significant direct effects

The accepted model after removing the non-significant paths is presented in Fig. 2.

Figure 2. shows that the direct effects in the final model. Goodness of fit values obtained from the final model have revealed that the final model was fit and acceptable (χ2 [246, N = 360] = 500,241; p = 0.00; χ2/df = 2,03; GFI = 0.90; AGFI = 0.87; NNFI = 0.90; CFI = 0.92; NNFI/TLI = 0.90; RMSEA = 0.05; SRMR = 0.06) [110–113]. Parameter estimates for the model are presented in Table 2.

**Table 2:** The direct effects of human basic values and constructive patriotism on active citizenship

Structural Relation			Direction	*β*	p
Constructive patriotism	→	Political literacy	+	0.24	***
Constructive patriotism	→	Participation	+	0.26	***
Constructive patriotism	→	Protest and social responsibility	+	0.27	***
Self-direction	→	Constructive patriotism	+	0.16	**
Universalism	→	Constructive patriotism	+	0.23	***
Power	→	Political literacy	+	0.14	**
Tradition	→	Political literacy	-	0.15	**
Stimulation	→	Participation	+	0.22	***
Benevolence	→	Participation	+	0.24	***
Stimulation	→	Protest and social responsibility	+	0.21	***
Self-direction	→	Protest and social responsibility	+	0.21	***

*p < 0.05, **p < 0.01, *** p < 0.001

According to the direct effect values in Table 2, constructive patriotism positively affected the sub-dimensions of active citizenship (constructive patriotism→ participation *β* = 0.26, *p* < 0.001; constructive patriotism→ protest and social responsibility *β* = 0.27, *p* < 0.001; constructive patriotism→ political literacy *β* = 0.24, p < 0.001). Also, self-direction and universalism directly positively affected constructive patriotism (self-direction →constructive patriotism *β* = 0.16, *p* < 0.01; universalism →constructive patriotism *β* = 0.23, *p* < 0.001).

When the direct effects of basic human values on active citizenship were examined, it was found that the power value, one of the self-enhancement values, directly affected the political literacy positively (power→ political literacy *β* = 0.14, *p* < 0.01). On the other hand, tradition, which is a conservative value, negatively predicted the political literacy dimension. (tradition→ political literacy *β* = -0.15, *p* < 0.01). Also, stimulation and benevolence directly affected the participation dimension positively, while the stimulation and self-direction directly positively affected protest and social responsibility dimension (stimulation→ participation *β* = 0.22, p < 0.001; benevolence→ participation *β* = 0.24, p < 0.001; stimulation→ protest and responsibility *β* = 0.21, *p* < 0.001; self-direction→ protest and responsibility *β* = 0.21, *p* < 0.001).

### Mediation analysis

In order to determine the significance level of the mediating effect of constructive patriotism, bootstrap 5000 resampling analysis was used in the study. Bootstrap analysis results are presented in Table 3.

**Table 3:** Indirect and total effects of the mediation models on active citizenship

Independent Variables	Mediator Variable	Dependent Variable	Indirect effects			Total effects		
			*β*	LCI	UCI	*β*	LCI	UCI
Self-direction	Constructive patriotism	Protest and social responsibility	0.04	0.016	0.080	0.25	0.148	0.352
Self-direction	Constructive patriotism	Participation	0.04	0.014	0.075	0.04	0.014	0.075
Self-direction	Constructive patriotism	Political literacy	0.04	0.013	0.073	0.04	0.013	0.073
Universalism	Constructive patriotism	Protest and social responsibility	0.06	0.026	0.107	0.06	0.026	0.107
Universalism	Constructive patriotism	Participation	0.06	0.024	0.100	0.06	0.024	0.100
Universalism	Constructive patriotism	Political literacy	0.05	0.021	0.097	0.05	0.021	0.097

LCI = Lower confidence interval (95%); UCI = Upper confidence interval (95%).

As can be seen in Table 3, the lower and upper confidence interval values obtained with the bootstrapping analysis do not contain the zero value (MacKinnon, et al., 2004). Therefore, it can be said that all mediation effects are significant. Self-direction has an indirect effect via constructive patriotism on protest and social responsibility (*β* = 0.04, 95% CI [0.016, 0.080]). Self-direction has an indirect effect via constructive patriotism on participation (*β* = 0.04, 95% CI [0.014, 0.075]). Self-direction has an indirect effect via constructive patriotism on political literacy (*β* = 0.04, 95% CI [0.013, 0.073]). Universalism has an indirect effect via constructive patriotism on protest and social responsibility (*β* = 0.06, 95% CI [0.026, 0.107]). Universalism has an indirect effect via constructive patriotism on participation (*β* = 0.06, 95% CI [0.024, 0.100]). Universalism has an indirect effect via constructive patriotism on political literacy (*β* = 0.05, 95% CI [0.021, 0.097]). The accepted and rejected hypotheses in the study are presented in Table 4.

**Table 4:** The accepted and rejected hypotheses in the study

Hypotheses		Result
H1	Universalism has a positive effect on participation.	Rejected
H2	Benevolence has a positive effect on participation.	Accepted
H3	Stimulation has a positive effect on participation.	Accepted
H4	Self-direction has a positive effect on protest and social responsibility.	Accepted
H5	Universalism has a positive effect on protest and social responsibility.	Rejected
H6	Stimulation has a positive effect on protest and social responsibility.	Accepted
H7	Tradition has a negative effect on political literacy.	Accepted
H8	Conformity has a negative effect on political literacy	Rejected
H9	Security has a negative effect on political literacy.	Rejected
H10	Power has a positive effect on political literacy.	Accepted
H11	Constructive patriotism has a positive effect on active citizenship.	Accepted
H12	Blind patriotism has no effect on active citizenship.	Rejected
H13	Self-direction has a positive effect on protest and social responsibiltiy via constructive patriotism.	Accepted
H14	Self-direction has a positive effect on participation via constructive patriotism.	Accepted
H15	Self-direction has a positive effect on political literacy via constructive patriotism.	Accepted
H16	Universalism has a positive effect on protest and social responsibiltiy via constructive patriotism.	Accepted
H17	Universalism has a positive effect on participation via constructive patriotism.	Accepted
H18	Universalism has a positive effect on political literacy via constructive patriotism.	Accepted

According to Table 4., H1., H5., H8., H9. and H12. numbered hypotheses were rejected. In contrast, H2., H3., H4., H6., H7., H10., H11., H13., H14., H15., H16., H17. and H18. numbered hypotheses were accepted.

### Discussion and conclusion

The current study found that constructive patriotism positively predicted all dimensions of active citizenship (political literacy, participation and protest, and social responsibility). This result is in line with previous studies [63–65]. For example, Schatz, et al. [65] found significant positive correlations between constructive patriotism and political effectiveness, political information gathering, and political activism. Also, constructive patriotism positively affects civic participation [63]. However, there are a few different findings [80].

Considering the relationship of basic values with patriotism, direct positive associations of universalism with constructive patriotism were found. This result is in line with the results of previous studies [81]. Because constructive patriots act for the welfare of their countries without giving up their universality value [17, 60, 81] similarly, those with universalism value act for welfare of all people [55]. Besides, in the current research, universalism related positively to all dimensions of active citizenship via constructive patriotism. The point to be underlined here in terms of Turkish culture is that universalism has no relationship with any dimension of active citizenship without the mediator role of constructive patriotism. Put another way, although universalism is a value that feeds activism, it needs the mediator role of constructive patriotism, which is an actional form of patriotism, in order to relate to active citizenship. As a matter of fact, universalism has associated with all dimensions of active citizenship via the mediator role of constructive patriotism. Because active citizenship is an action-oriented patriotism that includes questioning, constructive criticism, and being able to have opposite thoughts [65].

Self-direction, one of the values of openness to change, positively predicted constructive patriotism. Likewise, self-direction positively predicted all dimensions of active citizenship via constructive patriotism. This result is in line with previous studies. Because constructive patriotism is associated with the desire for change. Self-direction, which includes goals for change, relates to independent thought, civil liberties, change, and action at the core of constructive patriotism and active citizenship [55, 56, 60].

Stimulation was directly and positively related to the protest and social responsibility, and participation. This result is in line with previous studies. Because openness/extraversion values or personal characteristics and high education level positively affect participation in voluntary activities [114]. The motivational goals of the stimulation value are excitement, novelty, and challenge to life [55]. Because the individual who wants to be excited and can afford it can participate in the activities of non-governmental organizations or associations. In a sense, by challenging the existing conditions of life, it can challenge the impositions of the current order and participate in protest actions.

Benevolence was directly and positively related to the participation dimension. Because benevolence is associated with all voluntary behavior [58]. Also, benevolence includes goals related to welfare of all people. Since the motivational goal of benevolence value is to protect and increase the welfare of others [55], its positive effect on participation is theoretically expected. It has been stated in previous studies that benevolence values are associated with civic engagement [53, 56, 57]. Put another way, it is quite natural for the individual who aims for the welfare of others to participate in charity activities in his/her society as an active citizen.

Another significant result in the current research is that power directly and positively predicts political literacy. This result is similar to the findings of Luengo Kanacri et al. [53]. Luengo Kanacri et al. [53] found that those with higher levels of power-related values are more likely to be members of political organizations. Likewise, there are positive relations between power and free enterprise [55]. Because the motivational goals of the power value are to gain social status and prestige, as well as to establish dominance over people and resources and to control them. It can be said that in the study of Schwartz et al. [55], the goals of achieving social status and controlling other people came together with power and free enterprise. Likewise, in the present study, goals of social status and control over other people were combined with power and political literacy. This result becomes significant with a sociological and cultural evaluation. Because those who have political power in Turkish history and society have reached the highest status and thus have established control over other people [115]. Also, researches have shown that Turkish culture takes place in a collectivist vertical dimension that prioritizes hierarchy [94]. Therefore, it is quite meaningful that power predicts the political knowledge dimension for the purpose of gaining individual power. This result well reflected the Turkish culture and the theories on which the theoretical background of the research is based.

In the research, tradition value directly predicted political literacy negatively. Previous studies have found negative correlations between conservative values (tradition, conformity, security) and political activism [56, 58]. In the current study, conformity and security values did not predict political literacy. This difference may stem from the knowledge and action-based difference between political activism and political literacy. This result can be explained by the fact that individuals with tradition value do not need political knowledge because they do not have a purpose to change the current status quo [58, 81]. After all, tradition, which is a collectivist horizontal value, aims to have equality in society. The fact that it negatively affects political literacy, which is the first stage of acquiring status through political means, is entirely meaningful in terms of the theory [94].

### Practical implication

Based on the fact that education is the primary way to raise active citizens [14] and the scope and findings of the current study, values education has a critical importance starting from the preschool period. Globalization is more influential among young people in terms of behavior, attitude, and value changes [116] especially urban youth are more in touch with the effects of values such as independence, individual choice, and consumerism promoted by the global economy [117]. However, high value-similarity between emerging adults and their families through intergenerational value transmission [118] may make more functional values possible if their families are included in both non-formal and formal value education. Considering the prominent findings of the current study, it can be claimed that values education for openness to change (self-direction and stimulation) values is necessary for the protest culture, which is an essential indicator of active citizenship. Furthermore, a more culture-specific striking study result revealed that Turkish youth could only contact active citizenship with the value of universalism through constructive patriotism. In other words, activism towards global problems through universal values can only be possible with the help of constructive patriotism in Turkish youth. Therefore, constructive patriotism is a construct that should be considered in a values education program that will be integrated into the education system.

On the other hand, the current research findings indicated that promoting basic human values can be the initial stage of enhancing active citizenship self-efficacy. The research findings have revealed the importance of promoting stimulation and self-direction values from individualistic values and universalism and benevolence values from collectivist values. In this case, the stability of basic human values over time in Schwartz’s theory appears to pose a challenge in enhancing individuals’ active citizenship self-efficacy. However, contrary to the theoretical perspective, experimental studies conducted mainly with undergraduate students provided new insights by demonstrating that voluntary value change can occur through manipulation tasks [119]. In this context, interventions utilizing self-persuasion, consistency maintenance, and priming techniques can be employed to promote benevolence values among emerging adults. Furthermore, personal and contextual factors should be considered to strengthen manipulation tasks to promote self-transcendence values [120, 121]. For example, educational practices like volunteer experiences and service learning in schools or universities can play a significant role in promoting self-transcendence values [122].

### Limitations and future researches

Cross-sectional studies cannot provide causal relationships because they only involve obtaining the data to be analyzed from the target population at once, do not include follow-up and change over time, and measure outcome and exposure variables simultaneously. On the contrary, they can provide preliminary evidence for advanced research in the future and the discovery of associations between the variables [123]. Therefore, although hypothetical causality has been established in the current study, it is not possible to provide this with a cross-sectional design. In other words, the current study is neither longitudinal nor experimental. So it cannot yield conclusive [124] or deterministic causation but probabilistic causality through SEM techniques [109].

That said, research results have cultural characteristics. First of all, although cross-cultural validation of the basic human values scale used in the current research was conducted, cross-cultural validation of the patriotism scale was not conducted. Also, the fact that the active citizenship scale was developed in Türkiye limits the generalizability of the research in terms of different cultures. However, research provides transcultural information. For example, the critical requirement for constructive patriotism and active citizenship is autonomy, a trait that transcends culture. Although some cultural psychologists see non-Western people as having an interdependent self, the self-determination view suggests that being autonomous for individuals is a universal psychological need regardless of cultural differences [125]. In democratic governments, sharing power and paving the way for community well-being actions through civic participation impacts community dynamics directly. In this way, social cohesion can be achieved by activating social capital [126]. As a starting point, the quality of the relationships established by the parents in the family and the teachers at school will determine how the child exists in the social arena [125]. As a result, as in the current research, research results in social sciences may often vary according to the social context. Therefore, there is a need to examine the subject with an interdisciplinary approach, together with the life and cultural experiences of different societies.

The structural model proposed in the current study on the relationships between basic values, constructive patriotism, and active citizenship has beneficial outcomes for individuals, families, societies, and educational systems. A meaningful, purposeful, value-oriented, and socially beneficial life for the individual increases both subjective and communal/societal well-being. It creates a cyclical protective function between individual and public health. As social feeling, which is a sign of emotional health [7], attachment to the community and community satisfaction increase individual well-being positively [127]. Especially in eastern cultures, subjective well-being is related to harmonious homeostasis between the internal and external world of individuals [128]. For this reason, the relationships between active citizenship and individual well-being can be examined together in future studies.

## Data Availability

The datasets used and/or analysed during the current study are available from the corresponding author on reasonable request.

## Abbreviations

ACSES: Active citizenship self-efficacy scale
COVID-19: Coronavirus disease 2019
PAS: Patriotism attitude scale
PVQ: Portrait values questionnaire
SEM: Structural equation modeling
